# Unexpected stray attractors in confined leader-follower dynamics driven by cone-of-vision interactions

**DOI:** 10.1038/s41598-018-37457-y

**Published:** 2019-02-08

**Authors:** Amara A. Al-Sayegh, Sara A. Najem, Leonid Klushin, Jihad R. Touma

**Affiliations:** 1 Department of Physics, American University of Beirut, Riad El-Solh 1107, 2020 Beirut, Lebanon; 2 National Center for Remote Sensing, CNRS-L, Riad El Solh 1107, 2260 Beirut, Lebanon; 3 Beirut Research and Innovation Center, Chanteur Street, Ras al Nabeh, Beirut, Lebanon; 4 Institute for Macromolecular Compounds RAS, Bolshoi pr. 31, 199004 St. Petersburg, Russia

## Abstract

Experiments with groups of fish inside a circular tank have provided valuable insights into the nature of leadership in social groups. Sophisticated mathematical models were constructed with a view to recovering observed schooling and leadership behavior in such experiments. Here, and with the help of variations on a promising class of such models, we explore a dual set of social concerns, namely the likelihood of permanent evasion from a cohesive group by a controlled individual in confinement. Our minimal model reduces to a leader-follower configuration, with cone-of-vision driven interactions inside a circular domain. We show that the resulting dynamical system sustains a rich supply of non-aligned, straying “follower” states, the dynamics on which displays (chaotic) intermittency between boundary following behavior and infrequent long flights. We map these states in configuration space and explore transitions between them. We demonstrate robustness of observed behavior by considering model variations, as well as alternate leader control trajectory. While it is too early to draw the implications of leader-follower dynamics to collective behavior, we do confirm that a model stray fish relates to a self-organized school bouncing back and forth along the diameter very much like a follower responds to a point leader in our model. We further draw the implications of our results to the study of dynamical systems with discontinuities, robotics, and the study of human behavior in the face of normative control and confinement.

## Introduction

Self-organizing behavior of living organisms is ubiquitous. From the smallest to the largest scales, collective structures obtain and have received the attention of biologist, etiologists, sociologists, and increasingly physicists and mathematicians^1–5^. Phenomena range from bacteria swimming in formation, to swarming insects, to flocking birds, to schooling fish, to herds of wilder beast, to milling worshipers etc^6–9^. Controlled experiments whether on fish in tanks, or pedestrians in crowds are now added to a rich tradition of observational techniques deployed in the subject^10–13^. What is common to these works (and associated mathematical models) is an increasing focus on the nature of leadership in groups of animals moving in and out of coherent structures. Here, we identify novel formations of organisms in confinement and suggest a mechanism that gives rise to transitions between them. Our concern is not to mimic reality in its bewildering complexity. Instead, we explore the complexity of behavior sustained by simplified and reasonably grounded models, with a view to evaluating their relevance to one biological (and/or sociological) setting or the other. With this in mind, we adopt a promising recipe for alignment of fish as described in^10^ with the addition of interactions via a cone of vision as motivated in^13–15^. This model differs from novel recent minimal models which disregard the velocity-velocity alignment effect^16,17^ and whose universality is still debatable^15,18,19^. We then constrain organisms to move in a circular tank with a reflective boundary, having further split them into a *leader* bouncing on an invariant trajectory, and a *follower* which is responding to intermittent forcing by that *leader*. In pursuit of patterns sustained by our construct, we were lead away from popular concerns with the self-organization of a herd and its leaders, to the seeming inevitability of straying in confinement, and this despite a persistent will to align. We start with a brief description of our model, followed by an exhibit of typical behavior, then variations that test for robustness, and end by drawing implications of our results to a network of diverse fields.

“Fish” in our reference model are point particles with coordinates (*r*_*i*_, *θ*_*i*_), moving with a velocity vector ${\overrightarrow{v}}_{i}$, of constant magnitude ${\rm{v}}$, and varying direction *ϕ*_*i*_ as measured from the positive horizontal x-axis. They move inside a tank with a reflective circular boundary, centered at the origin of the coordinate system^10^. A model fish will interact and try to align with companions that are in its cone of vision which, at any given instant, is given by the minor circular sector enclosed between two rays cast symmetrically about its line of motion (along its instantaneous velocity vector), and the corresponding arc on the circular tank. We found it convenient to use *COV* to refer to both the cone of vision itself, as well as to the half aperature of that cone which can take values between 0 (no visibility) and *π* (full visibility). *COV*-mediated interaction is a biologically motivated alternative to topological interaction with Voronoi neighbors considered in^10^ [Note: In order to investigate information propagation during a leadership event in a fish school, Bayesian model selection was employed to test the marginal likelihood of four different models: metric, topological, Voronoi, and visual interactions^14^. The latter, which relies on the cone of vision algorithm described in the supplementary material of^14^, outperformed the others. It thus appears that significant information is provided by distinct neighbors, perhaps an adaptive strategy to dealing with the environment’s threats and uncertainties. This hypothesis is supported by substantial computational evidence in^13^]. A fish’s angular velocity *w*_*i*_ is adjusted according to the average behavior of members of the collective who happen to be in its *COV*:

1 $$\frac{d{w}_{i}}{dt}=-\,\frac{v}{\xi }({w}_{i}(t)-{w}_{i}^{\ast }(t)),$$

where,

2 $${w}_{i}^{\ast }=\frac{1}{{N}_{i}}\,\sum _{j\in CO{V}_{i}}\,\kappa \,\sin ({\varphi }_{j}-{\varphi }_{i}).$$

Here *ξ* is a persistence length scale, *COV*_*i*_ the cone of vision of fish *i*, ${w}_{i}^{\ast }$ the aligning social interaction term with coupling strength *κ*. Our *COV*-algorithm is a simplified version of the one in^14^, having dropped attraction and noise from the picture [see below for a noisy variation on the fiducial model]. Now comes a critical feature of our exercise which consists of further quenching dynamics down to two entities interacting in a leader-follower configuration. The leader moves along the vertical diameter of the circular boundary where it reflects elastically and is not affected by the follower. The follower tries to align with the leader whenever it is in its cone of vision and also reflects elastically from the circular enclosure [Note: In the context of schooling, the leader can be thought of as embodying the effective mean field of an organized group, a thought which we substantiate below, under subsection “The Leader as a group”]. The motion of the follower satisfies equations (1) and (2) with the leader being the only other particle it interacts with. We express these equations in dimensionless form, with the characteristic timescale $\tau =\frac{\xi }{v}$ (the time taken by the fish to adjust its angular velocity) and length scale R (the radius of the tank) taken as respective units. Thus the non-dimensional update equations for the angular velocity and the position of the follower *ω*(*t*) are now given by:

3 $$\begin{array}{rcl}\frac{dw}{dt} & = & -(w(t)-{w}^{\ast }(t))\\ \frac{d\varphi }{dt} & = & w(t)\\ \frac{dx}{dt} & = & {C}_{2}\,\cos (\varphi (t))\\ \frac{dy}{dt} & = & {C}_{2}\,\sin (\varphi (t))\end{array}$$

with

$${w}^{\ast }=\{\begin{array}{ll}{C}_{1}\,\sin ({\varphi }_{L}(t)-\varphi (t)) & {\rm{if}}\,{\rm{Leader}}\,{\rm{is}}\,{\rm{inside}}\,COV\\ 0 & {\rm{otherwise}},\end{array}$$

where *C*_1_ = *τκ* (the ratio of the time taken by the follower to change its orientation to the time it takes it to get affected by the leader), ${\varphi }_{L}(t)=\pm \,\frac{\pi }{2}$ and *ϕ*(*t*) are the orientations of the velocity vectors of the leader and the follower respectively and ${C}_{2}=\frac{v\tau }{R}$ (ratio of the time needed to change orientation to the time needed to cross with a distance equal to the tank radius *R*). The space of parameters is thus reduced to the *COV*, *C*_1_ and *C*_2_. In the rest of this paper, we consider *C*_1_ = 1 and *C*_2_ = 0.006 consistent with the values of *ξ*, *R* and *v* favored in^10^, and we vary the *COV* in the range 0 < *COV* < *π*, where *COV* = *π* represents full panoramic vision. Variations on this model are discussed in the “Robustness to Variations” section below [refer to the Methods section for details on the numerical treatment of Eq. 3].

## Results

### Attractors

Within this reduced phase and parameter space, we were surprised by the rich, unfamiliar, and to our mind highly significant behavior that remains possible. We had left ourselves with one single parameter *COV* with which to map out the dynamical evolution of one single member follower which is desperately seeking reunion with the group leader, while bouncing from the boundary of their mutual confinement. Varying that parameter, we explored random initial conditions for the follower on the boundary (the leader in these experiments is initially set at the base of the vertical diameter heading up), noting steady state behavior, to the best of our computational abilities and resources. A summary of typical behavior follows:

#### The Vertical Attractor

**Figure 1:**
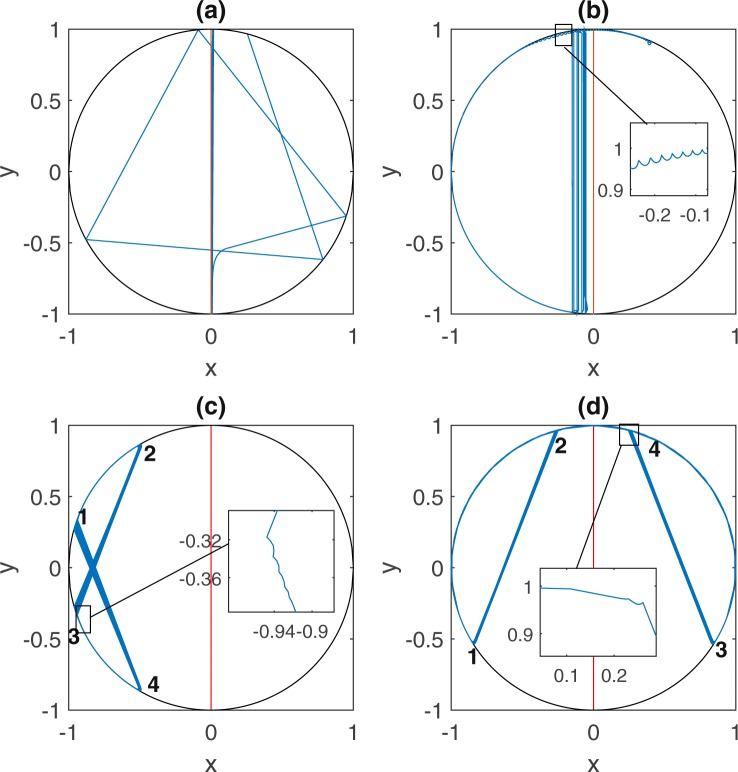
Attractors. Shown are samples of final states, with the leader (in red) and the follower (in blue): the vertical attractor in (**a** and **b**) (*COV* = 0.15 and *COV* = 2 respectively); the eight-figure attractor in (**c**) (*COV* = 1.6); the head-phone attractor in (**d**) (*COV* = 0.85). The insets in (**b**–**d**) elucidate the follower’s repeated short bounces near the boundary.

**Figure 2:**
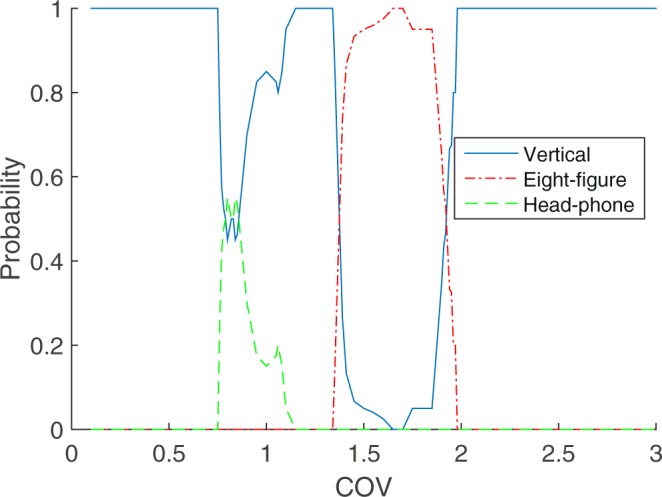
Phase Diagram. For each value of the *COV*, 50 trajectories with varying initial conditions are sorted. We display the probability of falling into each of the attractors described in the “Attractors” section. For *COV* < 0.75 the vertical attractor dominates in spite of the long time needed to get to it; for 0.75 < *COV* < 1.1 it coexists with the head-phone attractor which in its best situations attracts half of the trajectories; for 1.1 < *COV* < 1.34 the vertical attractor dominates again; for 1.35 < *COV* < 1.96 the probability of falling into the vertical attractor drops drastically in favor of the eight-figure attractor; eventually, for *COV* > 1.96 the vertical attractor dominates again by attracting all 50 trajectories.

Here the follower gradually approaches the leader on its diameter through a sequence of bounces off the boundary. Total alignment with the group, the outcome most thoroughly studied traditionally, is achieved asymptotically. One naturally expects that such alignment would obtain for a large enough *COV*. What one does not expect is that a blindingly small *COV*, coupled to the ever-present elastic boundary, promotes it equally well. For narrow *COV*, the follower hits the boundary at a spread of angles before it aligns with the leader and eventually gets attracted to it (Fig. 1a). For large *COV*, the follower directly aligns with the leader and asymptotically approaches the vertical diameter with each reflection at the wall (Fig. 1b). As evident in Fig. 2, alignment with the leader along its diameter is the favored outcome for *COV* < 0.75, 1.1 < *COV* < 1.34 and *COV* > 2.

#### The Eight-Figure Attractor

**Figure 3:**
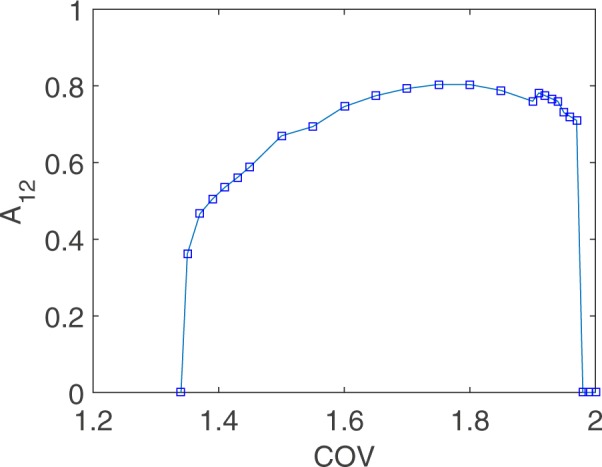
An Order Parameter for the Eight-figure. The orbit averaged angular separation *A*_12_ between vertices 1 and 2 of Fig. 1c provides a clean order parameter with which to map the eight-figure phase with increasing *COV*. We display the mean value of *A*_12_ over all cycles in a given trajectory, and over 50 trajectories with varying initial conditions, and the same *COV*. Near zero values of *A*_12_ indicate that the follower is hitting the upper (hence the lower) part of the circular tank at neighboring points, and that the follower is thus approaching the vertical attractor. Increasingly larger values of *A*_12_ indicate an increasingly well defined eight-figure attractor. Transition in and out of the eight-figure attractor is evident at *COV* ~ 1.3 and *COV* ~ 2 respectively.

A sample of this regime of entrapment is shown in Fig. 1c. A look at Fig. 2 reveals its presence for 1.35 < *COV* < 1.96 and its dominance for 1.5 < *COV* < 1.7. After a short transient, the follower is eventually trapped in an eight-figure which interleaves numerous short bounces by the follower (with the leader in sight after each bounce) and long range flights at points where the leader reverses direction at the boundary. Take-off and landing points (4 in total, making for the anchors of the eight-figure) fluctuate about mean vertices, with the cycle replaying while never exactly repeating. The transition in and out of this trap is neatly mapped by following the angular separation *A*_12_ between the mean position of neighboring anchors (1 and 2) as a function of the *COV*. As evident in Fig. 3, with increasing *COV* a transition point is hit beyond which *A*_12_ grows smoothly. With this transition, the vertical attractor gives way to an increasingly well defined eight-figure attractor. The latter dominates with larger *COV* till another sharp transition is hit, now resulting in a sudden drop in *A*_12_: the eight-figure trap unravels to the pull of an overwhelming leader, and and succumbs to efficient alignment into a vertical attractor.

#### Head-Phone Attractor

**Figure 4:**
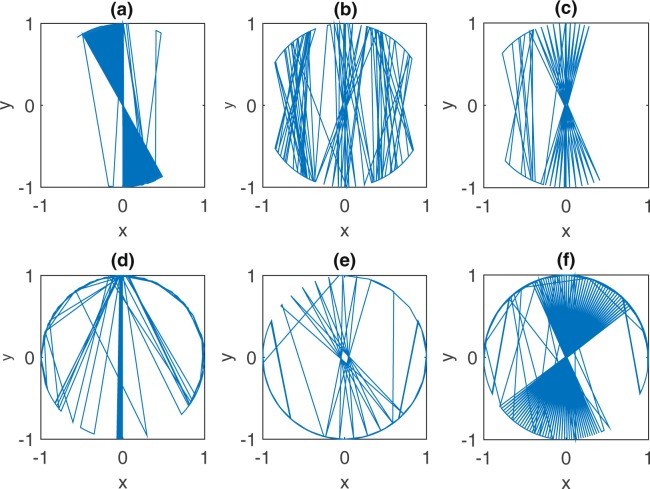
Behavior around Transitions. The follower enters into distinct transient regimes around transitions in and out of trapping states. In panels (a–c), the follower is shown near the critical point for transition to the eight-figure attractor (*COV* = 1.3, 1.31 and 1.32 respectively). The follower lingers over nearly central trajectories and alternates between right and left eight-figure attractors before settling on the vertical attractor for good. In panels (d–f), we show the follower’s transients trajectories near the critical points for emergence and disappearance of the head-phone attractor (*COV* = 0.75, 1.05 and 1.06 respectively). A similar pattern of lingering over near-radial orbits and alternation between attractors is observed.

The eight-figure attractor is not alone in trapping the straying follower. Another even more curious regime of control away from alignment appears for a narrow range of the *COV* (0.8 < *COV* < 1.1) (Fig. 2). We called it the head-phone attractor (Fig. 1d shows an example for *COV* = 0.85). Similar to the eight-figure attractor, it is repetitive without showing signs of relaxation into a periodic limit cycle. A strange attractor of sorts, its full (mathematical) structure remains to be unraveled. Its shape changes as a function of the *COV* while maintaining the distinctive feature of a follower spending much of its time tangent to the boundary. Close to the first transition point (*COV* ~ 0.8), the trajectory of the follower resembles the polygons of a classical circular billiard, before curving and falling into the head-phone attractor beyond transition. Around the second transition (*COV* ~ 1.1), the central trajectories of Fig. 4 are favored as transients.

In summary, and as synthesized in the phase diagram of Fig. 2, small and large *COV* favors the vertical attractor, while the head-phone and eight-figure attractor obtain over intermediate values of the *COV*. Not surprisingly the time scale for alignment with the vertical attractor is longest for the smallest *COV* and shortest for the largest *COV*. At transitions between vertical, head-phone, and eight-figure attractors, the follower lingers over numerous nearly central trajectories (Fig. 4) before settling on one regime or the other. These repeated excursions result in a spike of the time elapsed before settling into the ultimate attractor. We shall have more to say on this and related questions raised by the phase diagram in a forthcoming work.

### Robustness to Variations

The upshot of this overview of final states is that, within a rather minimal set of assumptions, one has a prevalence of non-aligned, straying states, over a respectable range of the control parameter, the *COV*. Importantly, this is not a freakish property of a very special model. Below, we briefly discuss intra- and inter-model variations on the results presented above, with the aim of assessing the robustness of those results.

#### Initial Conditions/Model Parameters

We explored a range of initial conditions for the follower inside the circle (not just at the boundary). This amounts to varying the initial phase shift for the follower relative to the leader. Transients aside, the same pattern of steady states emerged, as a function of the *COV*. The same is true of model parameters *C*_1_ and *C*_2_ albeit, with changes in the ranges over which those stray states obtain.

#### The Leader as a Group

Earlier in the introduction we claimed that the leader can be understood as the effective mean field of an organized group of fish. To support this claim, we generated a school of fish, all packed tightly while initially oriented along the diameter, and interacting with each other (as well with any stray member) following Eqs [1 and 2]. We report that such a school of *COV*-coupled fish follows the diameter while maintaining its cohesion through bounces, and that a straying member relates to the mean orientation of the school as a follower would relate to the orientation of the single particle in the position of the leader. So it would seem that we are justified in quenching internal degrees of freedom in a school, provided its members are held tightly enough along a diameter. What this admittedly encouraging outcome has to say about stray states in fully and freely interacting schools of fish is hard to tell, at least not before extensive simulations of such *COV*-coupled schools in confinement.

#### Leader Along the Rim

The tank’s circular boundary provides a natural periodic alternative to the diameter as a locus of the leader’s path. The follower’s response to a leader winding around the rim is qualitatively similar to the case with the leader on a diameter: the follower ends up aligned with the leader for small and large enough *COV*, with preference for landing on non-aligned states for intermediate *COV*. Non-aligned states include rotating variants of the eight-figure attractor.

#### Direct Follower Response

Here we perturb the model drastically by removing one dynamic degree of freedom: instead of the gradual relaxation of *w* described by equation (3) the equality $w={w}^{\ast }$ is imposed at all moments, equivalent to taking the persistence length *ξ* to zero. We expected to loose a number of the observed features in the process, given how the interplay between avoidance and alignment thrives on the gentle curving of the follower’s gaze together with sharp turn-around at the boundary. We were surprised to find the same qualitative outcome of follower landing on straying attractors (sharper versions of the head-phone and eight-figure attractors) for intermediate *COV* and aligning with the vertical attractor for *COV* small or large enough.

#### Noise and/or Boundary Softening

Last but not least, we wonder about the fate of the remarkable stray attractors when deterministic dynamics in our idealized model is perturbed by noise, and when specular reflection is replaced by a softer boundary-avoiding prescription. White Gaussian noise and a softer wall can be incorporated explicitly^10^, with *w*(*t*) now obeying a stochastic differential equation:

4 $$dw(t)=-\,\frac{v}{\xi }(w(t)-{w}^{\ast }(t))dt+\sqrt{C}N(t)\sqrt{dt}$$

where *C* is a measure of noise intensity, *N*(*t*) is chosen from a normal distribution with unit variance, and w^*^(*t*) is updated to include the effect of the wall as follows:

$$\{\begin{array}{ll}{w}^{\ast }=\frac{{k}_{w}}{{d}_{fw}}sign({\varphi }_{fw}) & if\,leader\notin COV\\ {w}^{\ast }=k\,\sin ({\varphi }_{L}(t)-{\varphi }_{f})+\frac{{k}_{w}}{{d}_{fw}}sign({\varphi }_{fw}) & if\,leader\in COV\end{array}$$

The constant *k*_*w*_ sets the reaction to the wall and has the meaning of a characteristic wall layer thickness; *d*_*fw*_ is the distance between the follower and the point *A* where its line of motion intersects the wall, and *ϕ*_*fw*_ is the angle between that line of motion and the normal to the surface of the tank at *A*.

With $\tau =\frac{\xi }{v}$, and *R* (the radius of the tank) taken as respective time and length scales, we rewrite the above equations in non-dimensional form

5 $$dw(t)=-\,(w(t)-{w}^{\ast }(t))dt+\sqrt{D}N(t)\sqrt{dt}$$

6 $$dx(t)={C}_{2}\,\cos (\varphi (t))dt$$

7 $$dy(t)={C}_{2}\,\sin (\varphi (t))dt$$

where w^*^ is:

$$\{\begin{array}{ll}{w}^{\ast }=\frac{{K}_{w}}{{d}_{fw}}sign({\varphi }_{fw}) & if\,leader\notin COV\\ {w}^{\ast }={C}_{1}\,\sin ({\varphi }_{L}(t)-{\varphi }_{f})+\frac{{K}_{w}}{{d}_{fw}}sign({\varphi }_{fw}) & if\,leader\in COV,\end{array}$$

with *D* = *τ*^3^*C*, *C*_1_ = *τk*, ${K}_{w}=\frac{\tau {k}_{w}}{R}$ and ${C}_{2}=\frac{v\tau }{R}$.

The follower is now subject to three coupled perturbations: the drive to align with the leader when in the COV, the randomizing effect of noise, and the soft turns induced by the wall. The three effects are controlled by the non-dimensional parameters *C*_1_, *D*, and *K*_*w*_ respectively.

**Figure 5:**
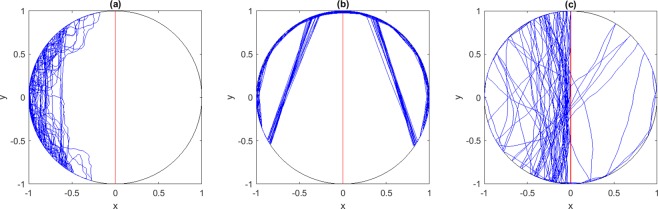
Eight-figure and Headphone Attractors with Noise Incorporated. Shown are samples of final states with the leader (in red) and the follower (in blue): (**a**) a diffuse eight-figure attractor (*D* = 0.05, *K*_*w*_ = 0 and *COV* = 1.6); (**b**) a not-so-sharply-defined head-phone attractor (*D* = 10^−5^, *K*_*w*_ = 0 and *COV* = 0.85); and (**c**) a dissolved head-phone attractors, with the follower ultimately aligning with the leader, after wandering repeatedly over head-phone like behavior (*D* = 0.001, *K*_*w*_ = 0 and *COV* = 0.86).

Keeping *C*_1_ = 1 and *C*_2_ = 0.006 as before, we explore for a start follower dynamics with specular reflection at the wall, in the presence of noise. Note that with our choice of the time-step *dt* = 10^−3^, the noise term is comparable to the maximum amplitude of the alignment term (assuming an initially straight trajectory) for the crossover value *D* = *D*_*cross*_ = 10^−3^. In order to better understand the expected noise effects, we notice that for a freely moving follower in the absence of the leader (w^*^ = 0) Eq. (5) describes a standard Ornstein-Uhlenbeck process^20^. For such a noise-modified free motion the mean-square angular deviation would asymptotically grow with time diffusively as $\langle {(\varphi (t)-\varphi (0))}^{2}\rangle =Dt$. Over the time required for a full tank traversal, $T=\frac{2R}{\tau v}=2/{C}_{2}$, the rms angular deviation would be given by $\delta {\varphi }_{rms}={(2D/{C}_{2})}^{1/2}$. Thus, at the crossover noise, *δϕ*_*rms*_ ≈ 0.58 which is of order one as expected. We sweep over *D* and report the following: **i**. for *D* as large as 10^2^ × *D*_*cross*_ traces of the eight-figure attractor are maintained; **ii**. for *D* = *D*_*cross*_ the eight-figure is maintained, but the headphone attractor is long lost; **iii**. for *D* smaller than 4 × 10^−5^ both the head-phone and the eight-figure attractors survive. Note that for *D* = 4 × 10^−5^ the magnitude of the noise term is about 20% of the alignment term, and the rms angular deviation over the tank traversal time is *δϕ*_*rms*_ ≈ 0.12. Samples of noisy eight-figure and headphone attractors, then an instance of a headphone lost to alignment with the leader, are shown in Fig. 5, displaying simulations with representative values of *D* at *K*_*w*_ = 0.

**Figure 6:**
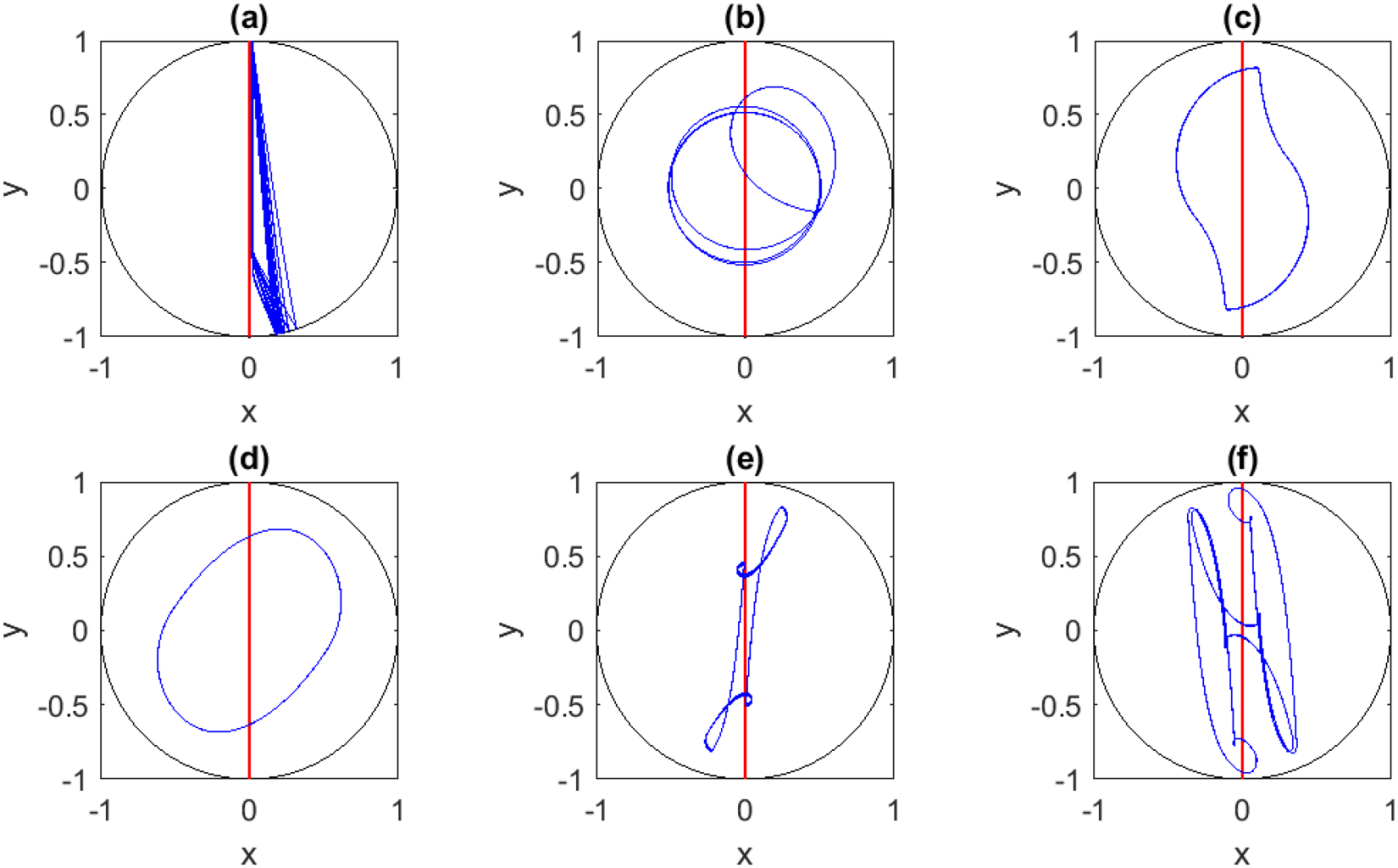
Novel Leader-Avoiding attractors. Shown are samples of final states, with the leader (in red) and the follower (in blue) with *D* = 0 and *COV* and *K*_*w*_ varying as follows: (**a**) *COV* = 0.45 and *K*_*w*_ = 10^−4^; (**b**) *COV* = 0.4 and *K*_*w*_ = 0.01; (**c**) *COV* = 0.4 and *K*_*w*_ = 0.01; (**d**) *COV* = 2 and *K*_*w*_ = 0.01; (**e**) *COV* = 1.6 and *K*_*w*_ = 0.1; and (**f**) *COV* = 2 and *K*_*w*_ = 0.1.

If, on the other hand, we set *D* = 0 and sweep over *K*_*w*_, attractors evolve in the following fashion: **i**. For *K*_*w*_ smaller than 3 × 10^−5^ both the headphone and eight-figure attractors survive; **ii**. In addition to that, for 4 × 10^−6^ < *K*_*w*_ < 3 × 10^−5^, a new leader-avoiding triangular attractor emerges having one side nearly parallel to the vertical attractor and a vertex opposite to this side on the circumference of the tank (Fig. 6 panel (a)); **iii**. for 3 × 10^−5^ < *K*_*w*_ < 10^−4^ the headphone attractor disappears while the eight-figure attractor survives; **iv**. for *K*_*w*_ > 10^−4^, new leader-avoiding attractors emerge, reflecting a tension between alignment and strong boundary avoidance. We show typical such attractors in panels b–f of Fig. 6.

**Figure 7:**
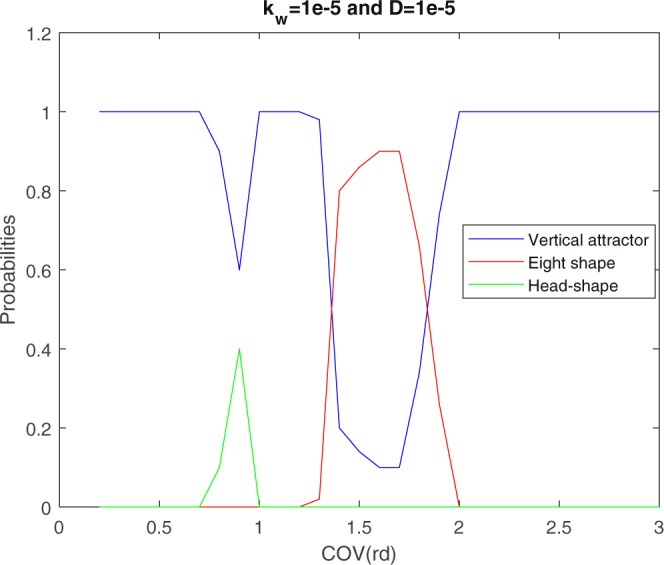
Phase Diagram with Noise and Boundary Softening Incorporated. For each value of the *COV*, 50 trajectories with varying initial conditions are sorted with *K*_*w*_ = 10^−5^ and *D* = 10^−5^. We display the probability of falling into each of the attractors described in the “Attractors” section. For *COV* < 0.7 the vertical attractor dominates in spite of the long time needed to get to it; for 0.7 < *COV* < 1 it coexists with the head-phone attractor which in its best situations attracts 40 percent of the trajectories; for 1 < *COV* < 1.3 the vertical attractor dominates again; for 1.3 < *COV* < 2 the probability of falling into the vertical attractor drops in favor of the eight-figure attractor; eventually, for *COV* > 2 the vertical attractor dominates again by attracting all 50 trajectories. All simulations were done with *dt* = 10^−3^.

Finally, and sweeping over *K*_*w*_ and *D* simultaneously, we find that both headphone and eight-figure attractors survive for *D* ≤ 4 × 10^−5^ and *K*_*w*_ ≤ 3 × 10^−5^. Allowing for moderate noise and soft reaction to wall with *D* = 10^−5^ and *K*_*w*_ = 10^−5^, we recover in Fig. 7 an analogue of the phase diagram of Fig. 2. Comparing both diagrams, we observe that the headphone and eight-figure leader avoiding attractors are maintained over the same intervals of *COV*, however the maximum probabilities for obtaining them are somewhat smaller in the presence of noise and boundary softening. Results in Fig. 7 are based on the final states of trajectories followed for 150 *T* (full tank traversal times). With long-term stability in mind, we followed straying trajectories for longer durations, confirming the survival of figure-eight attractors for as long as 3000 *T*.

We have here barely touched on the complex dynamical behavior ushered by noise and reaction to the wall. In addition to classifying novel attractors, their nature, origin and stability to perturbations in model parameters, one naturally worries about long term stability of attractors in the presence of noise. Whether stray attractors are only metastable (albeit with a very long lifetime) and whether noise-driven transitions between attractors may account for the experimentally observed intermittent behavior remain intriguing open questions which, along with those raised by the variations above, shall make for an interesting follow up to the present report.

For now, and having explored variations which tested extremes (whether in the leader’s trajectory, or the follower’s response), then allowed for noise and softer wall reaction, we can safely speak of a robust and relatively high probability of stray behavior over a broad range of *COV*. Try as we may, it seems that a follower confined within a reflecting boundary with a leader dictating alignment when in sight, remains with substantial freedom to stray when the cone of vision is broad enough for the boundary to help structure its motion away from the leader, and not too broad to find itself overwhelmed by that leader.

## Discussion

We were guided to the reported vault of unusual behavior by wondering whether communication through visual cues inside a closed circular domain can account for the observed alternation between coexisting self-organized phases in shoals of fish^12^. To be sure, our reduced leader-follower model is too crude to answer this question. Still, we find in its unusual phases, and transitions between them, an incitement to further explore visual communication networks. In fact, the *COV* was established as the most likely mode of information spreading in a fish school^13,14^. Variations in the *COV* are expected to occur naturally with the field of view of a given fish getting occluded by other shoal members or obstacles. We showed that such variations can lead to disruption of, if not shifts between attractors. We are thus tempted to ask: Could temporal variations in the effective *COV* of a given fish -variations which may result from the play of shadows and clearings in a shoal- lead to transitions from one self-organized state to the other and back?

On the other hand, and as already apparent from the presentation above, our results thrive at the interface between dynamical systems theory, systems control, and ultimately, and most interestingly, the study of human social behavior. We wish to conclude our exposition by briefly drawing out the connections and implications of our work in these fields.

The dynamics of a particle bouncing off a closed boundary belongs to the vast field of mathematical billiards^21–23^. It is not exactly the right place to review the literature. We simply point out the novel ways in which our system and associated results couple to the field: a- classically, a single particle is allowed to bounce off an enclosure of arbitrary shape; here we consider the controlling effect of a perturber/mean field confined with the particle: the resulting dynamics is no longer Hamiltonian, with strange attractors arising in the process; b- classically, when more than one particle are considered, it is with local collisional interactions in mind; here, we consider long range, intermittent interactions with the controlling agent; c- classically, the departure of the boundary from circular allows for Hamiltonian chaos; here dissipative irregular behavior over attractors emerges within a circular domain. The models we consider, and the behavior they sustain make for open mathematical questions which are best addressed within the growing field of dynamical systems with discontinuities^24,25^. There one learns that novel behavior arises when a particle hits the surface of discontinuities along a tangent, so called grazing orbits; here we have preliminary indications (Fig. 4) that the angle at which the particle encounters the boundary, how close it happens to be to the normal, plays a role around transitions from one attractor to another. Our exercise is complicated by the coupling between two sources of discontinuity, the reflective boundary, as well as visual encounters with the leader on its periodic trajectory. Anything more definite will have to await further experimentation, and careful mathematical analysis.

Pursuit-evasion problems^26^ come to mind in relation to our exercise. There the follower is programmed to head straight towards a moving target, with the design of intercepting it; here one asks about the possibility of eventual alignment with intermittent cone of vision contact. A history of rewarding developments in pursuit-type problems suggests interesting variations to ours which we intend to “pursue” actively. On another note, one wonders about strategies for guiding a self propelled particle into a given regime of motion, within confinement. Our work provides an intriguing answer: a diametric sentinel which dictates alignment of a robot through a broad enough cone of vision can force it into an eight-figure or head-phone trajectory, without the need for continuous, active, monitoring.

It is straightforward to link our model and the non-aligned states which it sustains with physically inspired modeling of opinion dynamics, particularly when a collective is caught between the competing influence of media on one large scale, and that of neighboring social groups on another more local scale, with the undecided group playing a crucial role in the outcome^27^.

We would like to conclude the discussion section by drawing a provocative connection (deliberately speculative to be sure, but exciting nonetheless!) to a set of dynamically flavored observations by Milgram and Goffman, respectively. Works by both social scientists have inspired much mathematical modeling of social dynamical phenomena, with Milgram’s pioneering work on the “small world phenomenon”^28^ stimulating extensive studies of network(ed) dynamics (e.g.^29^), and Goffman’s insights into pedestrian traffic^30,31^ coupling to recent studies of crowd dynamics in public spaces^7^. Here, it is through their independent work on human response to conflicting pressures to conform, that we see them further informing socio-dynamical modeling^31–33^. The connection was largely stimulated by the explicitly dynamical language employed by both Milgram and Goffman, in settings which we believe are germane to ours. [Note: it goes without saying that highlighting affinities between our work and the social insights of Milgram and Goffman is a long way from implying anything secure about the connection which would require extensive modeling of related socio-dynamical settings. The later is of course beyond the scope of our humble contribution]. In experiments probing “the conditions of obedience and disobedience to authority”^32^, Milgram’s subject is caught between obedience to authority and compassion with the victim. Milgram notes variations in the subject’s response with closeness of authority, and proximity to the victim, describing the two influences as fields of force that weaken with distance. Milgram notes the tension between the two influences, promoting erratic behavior in the subject, all the while being trapped in a conflicting state without the sufficient strength to terminate the experiment. Similarly, the particle in our experiments is caught between the leader’s normative (but intermittent) influence and the boundaries reflective perturbation, and finds itself with sufficient (though not overwhelming) strength/presence of authority, trapped in shall we say *un*-*willfull states of incomplete obedience*. With our follower’s predicament in mind, one wonders how Milgram’s results would have come out with intermittent rather than persistent intervention by authority, whether close or far. Equally suggestive, and perhaps more tempting to dialogue with, is Goffman’s extensive treatment of an individual’s self as shaped (even constituted) by continuous dynamical interactions with the social environment^34^. Goffman was keenly interested in mapping the force fields (often times mediated by face-to-face interactions) which couple the individual to authority. For Goffman, a “gathering” is that interactive, confined and confining space in which a collective is self-regulated through those dynamically shifting force fields. Goffman notes that in a given “gathering”, the individual is constrained to maintain a consistent, “viable image in the eyes of others”. He further notes that due to the constant, and unexpected shift in local circumstances, adjustments will be continuously necessary, to the point that an individual’s sense of him- or her-self emerges as a dynamical byproduct of inter-face with fluctuating social encounters^31^. Could the trapping attractors of our follower in relation to a capricious leader, re-emerge, in a more sophisticated setting, as dynamical analog’s of Goffman’s “conception of the self as contingent in the sense of being only probabilistic, a theatre run of performances to audiences and critics whose responses are always in play, whose applause is never certain”?^33^ The curtain is drawn at this speculative note.

## Methods

Equations 3 are solved using an Euler scheme with time step *dt* = 0.001: the position and orientation of the follower are integrated forward in time, while the leader moves unaffected along the vertical diameter, with the same speed as the follower. The leader’s position relative to the follower’s cone of vision is tracked in order to update ω^*^ in Eq. 3.

Collisions with the boundary of both the follower and leader are treated as follows: when the updated particle’s position falls outside the boundary with a 10^−10^ margin the particle is reflected back into the circle. More precisely, we define ${\overrightarrow{v}}_{b}$ to be the velocity vector just before collision, ${\overrightarrow{r}}_{0}$ to be the vector joining the center of the circle and the point of intersection of ${\overrightarrow{v}}_{b}$ with the boundary, and ${\overrightarrow{v}}_{a}$ be the velocity vector after collision, which is shown to be:

8 $${\overrightarrow{v}}_{a}={\overrightarrow{v}}_{b}-2({\overrightarrow{v}}_{b}\cdot {\overrightarrow{r}}_{0}){\overrightarrow{r}}_{0}$$

Then we can write:

9 $${\varphi }_{a}=\arctan \,(\frac{\sin (2{\theta }_{0}-{\varphi }_{b})}{\cos (2{\theta }_{0}-{\varphi }_{b})}),$$

where *ϕ*_*a*_, *ϕ*_*b*_, and *θ*_0_ are the angles the vectors ${\overrightarrow{v}}_{a}$, ${\overrightarrow{v}}_{b}$, and ${\overrightarrow{r}}_{0}$ make with the positive *x*–*axis* respectively. Additionally, to balance out the traveled distance outside the boundary the position is updated as follows:

$$\begin{array}{l}{x}_{a}={x}_{0}+c\,\cos \,{\varphi }_{a}\\ {y}_{a}={y}_{0}+c\,\sin \,{\varphi }_{a},\end{array}$$

where $c=x^{\prime} \,\cos \,{\varphi }_{b}+y^{\prime} \,\sin \,{\varphi }_{b}-\sqrt{1-{(x^{\prime} \cos {\varphi }_{b}-y^{\prime} \sin {\varphi }_{b})}^{2}}$, and (*x*_*a*_, *y*_*a*_), (*x*′, *y*′) and (*x*_0_, *y*_0_) are the particle’s coordinates before collision, outside the circle and those of ${\overrightarrow{r}}_{0}$ respectively. For each value of *COV* 50 instances with varying initial conditions were followed for 5 × 10^7^ integration steps which amounts to 150 full tank traversal times. The results, which depend on initial conditions, are then summarized in the phase diagram.

## Data Availability

The datasets generated during and/or analyzed during the current study are available from the corresponding author on reasonable request.
